# Mature dendritic cell derived from cryopreserved immature dendritic cell shows impaired homing ability and reduced anti-viral therapeutic effects

**DOI:** 10.1038/srep39071

**Published:** 2016-12-13

**Authors:** Qianqian Zhou, Yulong Zhang, Man Zhao, Xiaohui Wang, Cong Ma, Xinquan Jiang, Tao Wu, Donggen Wang, Linsheng Zhan

**Affiliations:** 1 Beijing Institute of Transfusion Medicine, Beijing key Laboratory of Blood Safety and Supply Technologies, Beijing 100850, P.R. China; 2 Key Laboratory of Advanced Energy Materials Chemistry (Ministry of Education), Nankai University, Tianjin 300071, China; 3 School of Public Health, Taishan Medical University, Taian, Shandong 271000, China; 4 Department of Blood Transfusion, PLA Army General Hospital, Beijing 100700, P.R. China

## Abstract

Cryopreservation is critical in reducing redundant operations and also in quality control in dendritic cell (DC) therapy. Full maturation and efficient homing of DCs to T cell-region constitute a crucial aspect of DC immunotherapy; however, the *in vivo* migration and distribution pattern, as well as the anti-viral effect of DCs that matured from cryopreserved immature DCs (cryoim-mDCs) remain to be revealed. In the present study, we compared cryoim-mDCs with DCs matured from fresh immature DCs (fmDCs) in the aspects of phenotypes, *in vivo* homing capacities as well as the anti-viral therapeutic effects to further clarify the effect of cryopreservation on DC-based cytotherapy. The results showed that cryopreservation impaired the homing ability of DCs which was associated with the reduced expression of CCR7 and disturbed cytoskeleton arrangement. Moreover, the antigen-specific CD8+ T cell response induced by cryoim-mDCs was much weaker than that induced by fmDCs in both the spleen and liver draining lymph nodes, which provided reduced protection from viral invasions. In conclusion, cryopreservation is a good method to keep the viability of immature DCs, however, the *in vivo* homing capacity and anti-viral therapeutic effect of DCs matured from frozen immature DCs were hindered to some extent.

As the most potent professional antigen-presenting cells (APCs), dendritic cells (DCs) bridge the gap between the innate and adoptive immune responses and are the only ones capable of priming naïve T cells^1^. DCs can be divided into two heterogeneous subsets according to the development stages they experience, *i.e.*, immature and the mature DC. Immature DCs (imDCs) effectively capture and process antigen but limitedly express allostimulatory molecules (CD40, CD80 and CD86), whereas mature DCs (mDCs) can be generated from imDCs by stimulations from toll-like receptors (TLRs) and have a higher expression of costimulatory molecules as well as an elevated ability to home to lymph nodes (LNs)^2^.

The capability of mDCs to generate antitumor or anti-viral immune response both *in vitro* and *in vivo* has been documented in many disease models. In these experiments, imDCs were usually isolated *in vitro* and loaded with tumor or viral antigens and matured by adjuvants to become mDCs, and these antigen-bearing mDCs were then injected into syngeneic animals as anti-cancer or anti-viral vaccines^3^,^4^. To date, DC-based immunotherapy has been tested on small cohorts of advanced cancer patients, who had failed to respond to conventional therapies, and increasing clinical trials are underway, however, only a fraction of these patients showed vaccine-induced immune responses and an even small proportion (10–15%) exhibited a clinical response^5^,^6^. Among those major factors resulting in the failure of adoptive DC therapy to induce sufficient acquired immunity, the percentage of injected DCs that migrated from the injection site to the draining lymph nodes is believed to be a critical limiting one^7^.

Enormous animal studies and clinical trials have proved repetitive administration of DCs is important to achieve clinically relevant T cell responses^8^. However, the time-consuming and cost-intensive procedure in the generation of DCs as well as the batch-to-batch variations dramatically limit the feasibility of repeated vaccinations. That to produce sufficient numbers of DCs at one time point and then cryopreserve them in aliquots ready for clinical application would dramatically improve the practicability of DC-based vaccination^9^. Hence, the properties of cells that have experienced freezing-thawing cycle need to be fully addressed. Several studies in the early 2000s and recent years have described the effect of cryopreservation on the biology and function of DCs *in vitro* or *in vivo*, however, some conclusions are controversial^10^,^11^,^12^,^13^ and most importantly, up to now, there is still a lack of confirming evidence on whether cryopreservation would have an effect on DCs’ homing capacity.

In the present work, we focused our study on the comparison between mature DCs derived from fresh imDCs (fmDCs) with that from the cryopreserved imDCs (cryoim-mDCs) in the aspects of phenotypes, *in vivo* homing capacities as well as the anti-viral therapeutic effects to clarify the effect of cryopreservation on DC-based immunotherapy. The evaluation of their homing capacities was carried out by bioluminescence imaging method (BLI). As an emerging *in vivo* cell tracing method, BLI has the advantages of high specificity and sensitivity and most importantly, it can visualize cells’ dynamic migrating process by successive imaging^14^,^15^. Thus, it could provide us more detailed and objective information about DCs’ homing process before and after cryopreservation. In addition, we also highlighted the relevance of DC location to the intensity of the antigen-specific T cell responses that elicited. We believe the elucidation of the influences of cryopreservation on the spatiotemporal dynamics of DC migration *in vivo*, and the correlation analysis between DC homing and the elicited therapeutic effects would greatly attribute to the optimization of DC vaccine’s preparation and then to minimize the adverse influences of the freezing-thawing process.

## Results

### Cell viability and immunophenotype analysis

**Figure 1:**
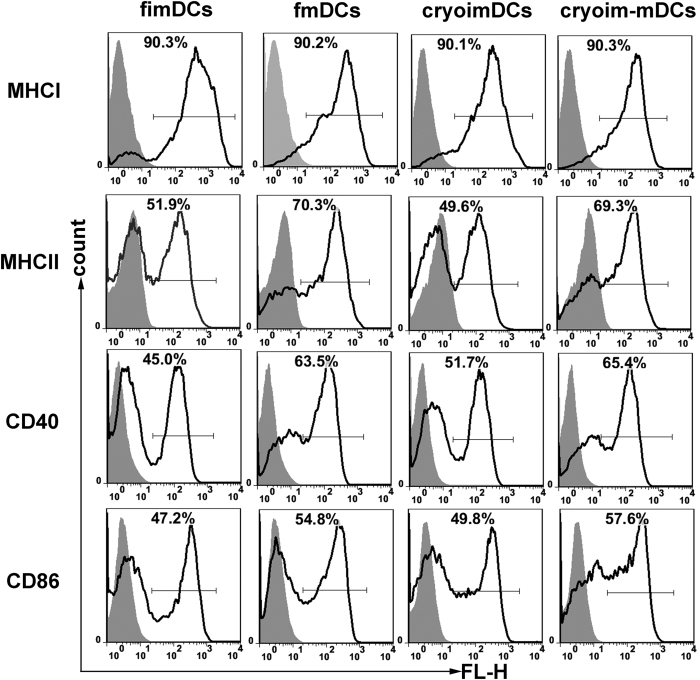
Cryopreservation had little effect on DCs’ phenotype marker expression. The fimDCs, cryoimDCs, fmDCs and cryoim-mDCs were collected and doubly stained with APC-CD11c and one of FITC-MHCI, FITC-MHCII, PE-CD40 and FITC-CD86, respectively. The positive gate and specificity of these antibodies was controlled by corresponding isotypes.

There were four types of DCs involved in this study, that is, fresh immature (fimDCs), cryopreserved immature DCs (cryoimDCs), fmDCs, and cryoim-mDCs. The relationship among them was described in detail in Figure S1 in Supplementary Materials. These cells were subjected to fluorescence activated cell sorter (FACS) analysis to examine the effect of cryopreservation on cellular viability and the surface marker expressions. Total cell populations (R1) in FACS analysis were determined by light scatter properties with the purpose to include most of cells and excluded the debrises. The population of DCs (R2) was defined by CD11c positive population of R1 (Figure S2A in Supplementary Materials). To examine cell recovery rate, imDCs before and after cryopreservation were stained with PI and Annex-V and then were detected by flow cytometry. The viability of cryopreserved cells was well kept and a recovery rate of about 90% was obtained (Figure S2B in Supplementary Materials). Data from phenotype marker detection showed that the molecules of CD40, CD86, MHC I and MHC II on the fimDCs and cryoimDC were expressed at comparable levels, which means that surface markers on imDCs didn’t change upon freezing-thawing process (Fig. 1). We also compared the viability and surface markers expressions of cyroimDCs between 0 h and 24 h after the thaw of the cells (Figure S3 in Supplementary Materials), demonstrating that the extension of the cultivation time of cryoimDCs only have a slight effect on their viability or maturation. In addition, when fimDCs or cryoimDCs were matured by LPS, both fmDCs and cryoim-mDCs exhibited significant allostimulatory markers elevation (CD40, CD86, and MHC II) compared with their immature counterparts and no difference was observed between these two groups, suggesting cryopreservation didn’t affect imDC maturation in the aspect of phenotype marker elevation. Different from CD40, CD86, and MHC II, the MHC I molecule was constitutively expressed on those four types of DCs with a very high positive percentage (≈90%) and was not affected by the cryopreservation or stimuli signal.

### The homing ability of subcutaneously injected fmDCs and cryoim-mDCs

**Figure 2:**
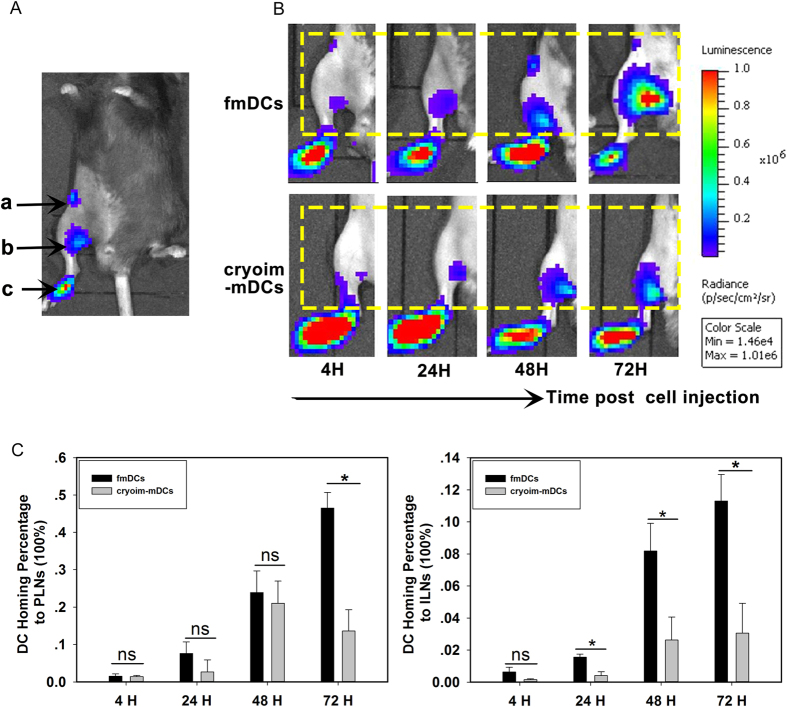
Comparing the homing ability of subcutaneously injected fmDCs and cryoim-mDCs. A total of 1 × 10^6^ L2G85.C57BL/6 derived DCs were injected subcutaneously in the hind leg footpad of C57BL/6 mice and were imaged successively at 4, 24, 48 and 72 h to reflect cells’ dynamic migration process. Mice were imaged for one minute under anesthesia. (**A**) Annotation on the source of lights from Fluc+ DCs after footpad injection. a: inguinal lymph nodes (ILNs); b: popliteal lymph nodes (PLNs); c: footpad (injection position). (**B**) The dynamic homing process of fmDCs and cryoim-mDCs. (**C**) Statistical data of cell-percentage homing to PLNs and ILNs. Data are expressed as mean ± SD (error bars). n = 5; ns, not significant; *p < 0.05 by Student’s t-test.

Having shown that there weren’t significant changes of allostimulatory molecule expression between fmDCs and cryoim-mDCs, we further subcutaneously injected DCs into the footpad of mice to examine their homing ability to draining lymph-nodes by BLI. Figure 2A showed the annotation on the source of light. The signal intensity in region “a” represented the number of DCs that migrated to inguinal lymph nodes (ILNs), region “b” represented the ones that migrated to popliteal lymph nodes (PLNs) and region “c” represented the residual DCs in footpad. Mice were imaged at 4 h after injection, as well as 24, 48 and 72 h later (Fig. 2B). Data showed that fmDCs began to migrate to the PLNs as early as at 24 h post injection and then to the ILNs at 48 h. Cryoim-mDCs also could be detected with visible signals from ILNs at 24 h post injection (Fig. 2B). However, the signal intensity from either PLNs or ILNs of cryoim-mDCs injected mice was much weaker than that of fmDCs group. Light emitted can be quantified within a delimited region by Living Image software (Fig. 2C). The percentage of ILNs and PLNs homing DCs were calculated according to the formula I_DCs_ = a/(a + b + c) and P_DCs_ = b/(a + b + c), respectively. The result showed that the homing percentage of fmDCs to PLNs at 72 h was 0.465 ± 0.042, which was more than 3-fold higher than that of cryoim-mDCs (0.136 ± 0.057). In addition, the ILN-homing cell percentage of fmDCs at 24, 48 and 72 h was also significantly higher than that of cryoim-mDCs. We also examined the *in vivo* homing ability of fimDCs and cryoimDCs (Figure S4 in Supplementary Materials). Statistical data showed that there weren’t distinct differences between fimDCs and cryoimDCs in homing to LNs and most of them remained confined to the footpad at all examined time points, suggesting the free-thawing process didn’t alter the migratory capacity of imDCs.

### The *in vivo* distribution pattern of intravenously injected fmDCs and cryoim-mDCs

**Figure 3:**
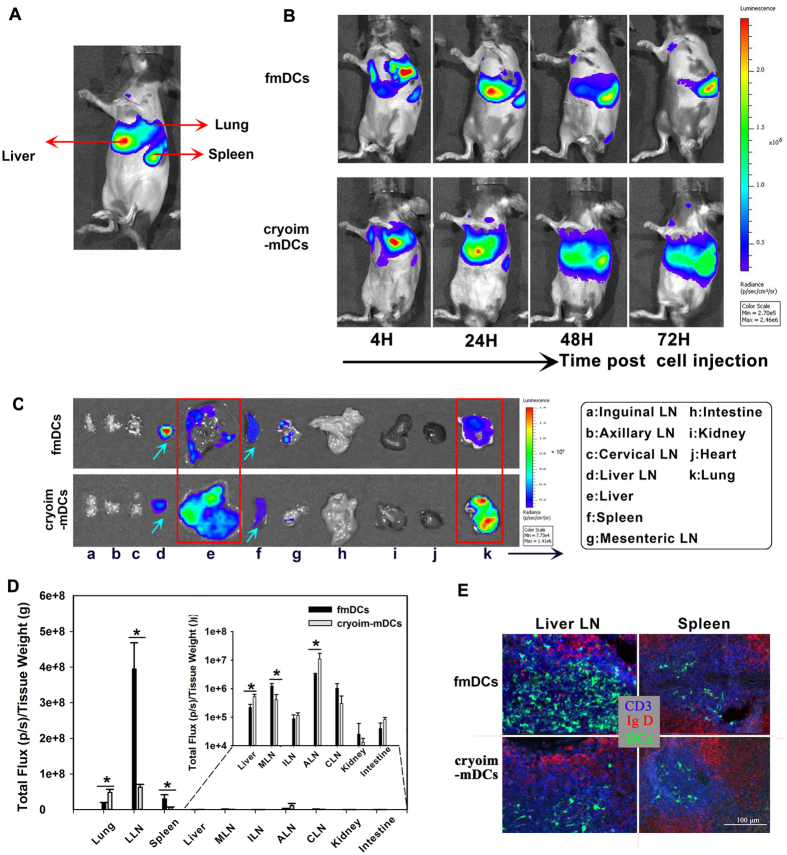
Comparing the homing and distribution of intravenously injected fmDCs and cryoim-mDCs. A total of 3 × 10^6^ L2G85.C57BL/6 derived DCs were injected intravenously into the recipients and cells’ dynamic migration and distribution process was detected. (**A**) Annotation on the source of lights from Fluc+ DCs after *i.v.* injection. (**B**) The dynamic homing process of fmDCs and cryoim-mDCs in living animal. (**C**) Tissue-distribution of fmDCs and cryoim-mDCs. (**D**) Statistical analysis of tissue distribution of DCs. The total amount of Fluc activity was normalized to the weight of the individual tissue. The data are presented as the mean ± SD (n = 5); *p < 0.05 by Mann-Whitney U test. (**E**) Immunofluoresence (CD3, blue; IgD, red; DC, green) of the spleen and LLNs at 72 h after GFP^+^ DC injection.

In contrast to the subcutaneous way of vaccination which directs DC vaccines to local lymph nodes, the intravenous administration is also widely used to deliver DCs to multi-lymphoid organs. *Ex vivo* generated Fluc+ fmDCs and cryoim-mDCs were injected intravenously into recipient mice for kinetic imaging analysis. Figure 3A showed the annotation on the source of light. A similar cell distribution pattern was found among these two groups at 4 h after cell injection; that is, large amounts of injected DCs accumulated in the lungs, with a small percentage in the liver or spleen (Fig. 3B). However, the trafficking discrepancy between fmDCs and cryoim-mDCs began to appear in the sub-sequential time points by dynamic detection. fmDCs began to emigrate from the lungs since 24 h, and were mainly concentrated in the spleen at 72 h. By contrast, the emigration of cryoim-mDCs was obviously impaired and a large amount of them could still be detected in the liver and lungs at 72 h.

Because it is difficult for light to emit from deeper and smaller tissues, we then performed a tissue bio-distribution analysis at 72 h after cell injection to evaluate DC present in various tissues that listed in Table S1 in Supplementary Materials. Figure 3C showed the representative tissue distribution data for DCs. For both fmDCs and cryoim-mDCs, no detectable or very weak signals were measured in the kidneys (i), intestines (h), hearts (j), inguinal lymph nodes (a), axillary lymph nodes (b), cervical lymph nodes (c) and the mesenteric lymph nodes (g) at 72 h post cell injection. The lungs (k) and the liver (e) are identified as two major organs for cryoim-mDCs to locate in, which was consistent with the data acquired from the living animals (Fig. 3B). By contrast, for fmDCs, light signals emitted from the liver or lung were very weak, whereas, the liver lymph nodes (LLNs, d, framed by a blue rectangle) and spleen were identified as the most predominant organs for DC accumulation. To better highlight the relative tissue distribution of DCs, the Fluc activity in those tissues was quantified and normalized to the weight of the individual tissue (Fig. 3D). Compared to fmDCs, cryoim-mDCs exhibited a significantly lower Fluc activity in the lymphoid tissues of LLNs, Mesenteric lymph nodes (MLNs) and the spleen, accompanied with statistically higher values in the lung and liver. These data suggested that in addition to the way of subcutaneous administration, the lymphoid-tissue homing ability of intravenously transfused cryoim-mDCs was also significantly lower than its fresh counterparts, which was further confirmed by the immunofluorescence data from the spleen and LLNs (Fig. 3E).

### The chemokine-receptor expression and cytoskeleton organizations of fmDCs and cryoim-mDCs

**Figure 4:**
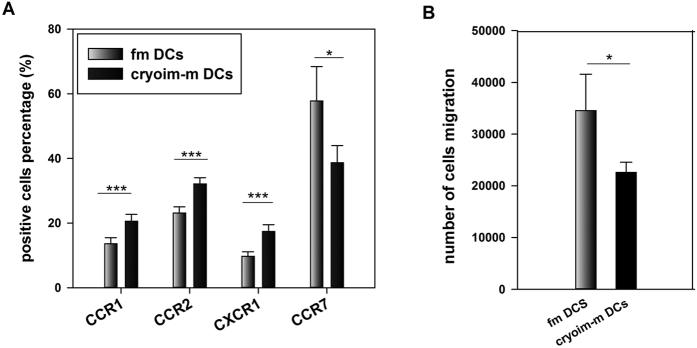
The chemokine receptor expression and chemotaxis of DCs. (**A**) fmDCs or cryoim-mDCs were doubly stained with APC-CD11c and one of PE-CCR1, PE-CCR2, PE-CXCR1 and PE-CCR7 respectively. The gate and specificity of these antibodies was controlled by corresponding isotypes. Data are expressed as mean ± SD; n = 5; *p < 0.05; ***p < 0.001 by Student’s t-test. (**B**) Chemotaxis of fmDCs or cryoim-mDCs was assessed by their migration through 8 *μ*m polycarbonate membrane using CCL19 as the chemoattractant. The migrated cells were counted and the results were expressed as the mean number of migrating cells ± SD; n = 5; *p < 0.001 by Student’s t-test.

In order to investigate the mechanisms for the declined homing capacity of cryoim-mDCs, we compared the expressions of CCR1, CCR2, CXCR1 and CCR7 on fmDCs and cryoim-mDCs by FACS. The positive percentage of CCR1, CCR2 and CXCR1 were significantly higher on cryoim-mDCs, whereas CCR7 was obviously lower on them (Fig. 4A). The mean fluorescence intensity (MFI) of all those chemokine-receptors except for CCR2 shared the same tendency with the detection of the percentage of positive-cells (Figure S5 in Supplementary Materials).

The trans-well experiment was then employed to evaluate DC chemotaxis towards CCL19 and a significantly decreased cell number of cryoim-mDCs was observed to pass through the 8 *μ*m membrane, which was in favor of the lower expression of CCR7 on them (Fig. 4B).

**Figure 5:**
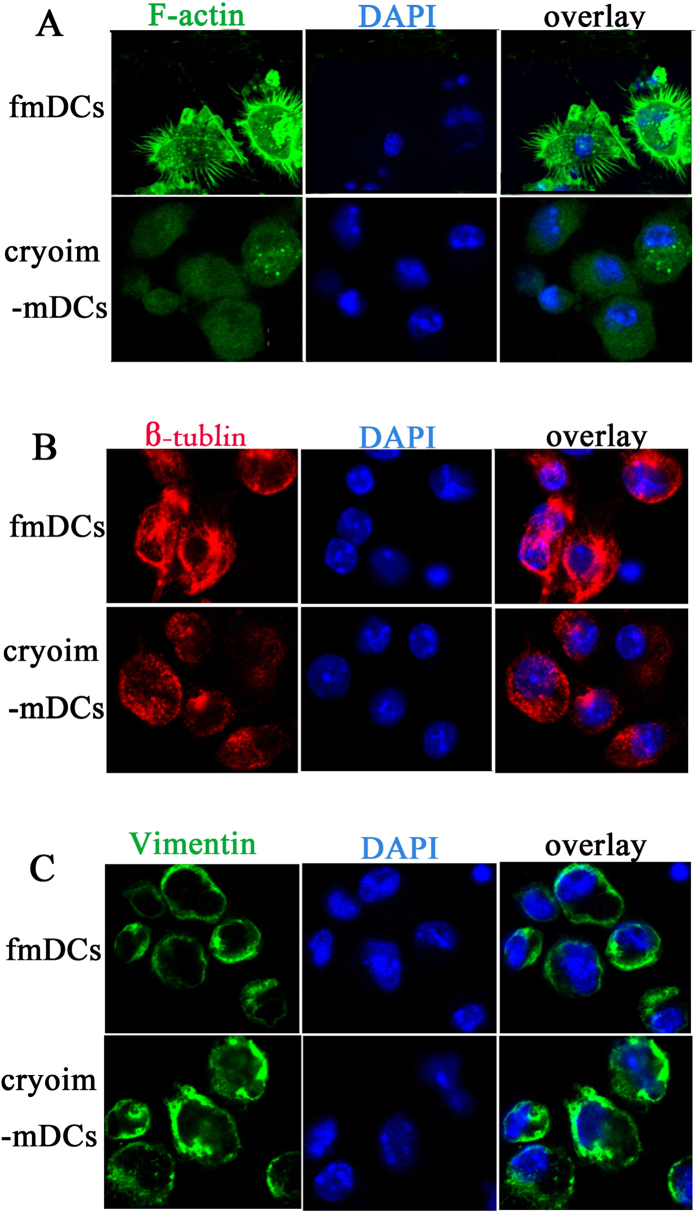
The cytoskeleton organizations of fmDCs and cryoim-mDCs. Cells were labeled with FITC-conjugated phalloidin, anti-*β*-tublin followed by Cy3-Goat Anti-Mouse IgG and anti-vimentin followed by 488-Alex-Goat Anti-Rabbit IgG, respectively, to visualize the organization of microfilaments (**A**), microtubules (**B**) and intermediate filaments (**C**).

We also performed an immune-fluorescence analysis of F-actin, microtubules and intermediate filaments in DCs (Fig. 5). F-actin staining showed that the fmDCs had a strong staining at cellular margin and dendrites while cryoim-mDCs exhibited a disappearance of F-actin positive dendrites and a much weaker marginal staining (Fig. 5A). The staining of *β*-tubulin showed that the microtubules in fmDCs were well-organized and appeared as fibers spreading radially out from the center of cells, by contrast, the organization of the *β*-tublin cytoskeleton in cryoim-mDCs was disrupted and became diffused (Fig. 5B). We did not find obvious differences between fmDCs and cryoim-mDCs in the intermediate filaments by the staining of vimentin (Fig. 5C).

### The antigen-specific CD8+ T Cell Immune Responses and anti-viral effect of fmDCs and cryoim-mDCs

**Figure 6:**
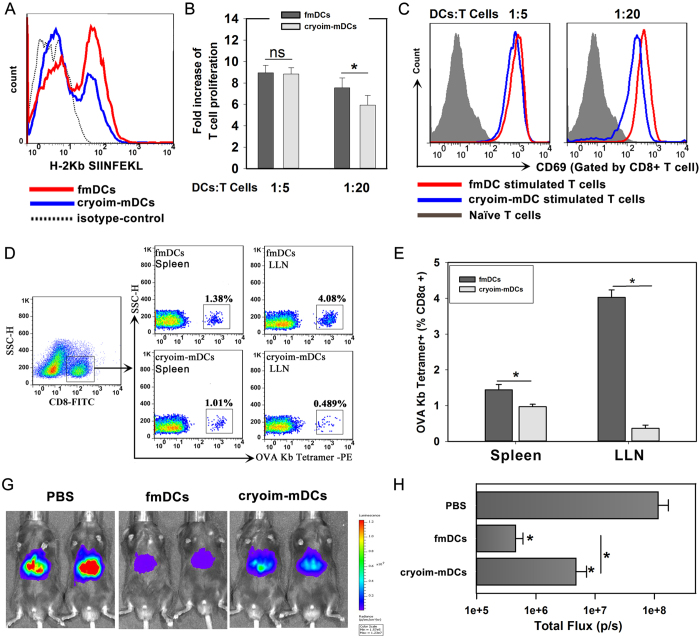
The antigen-specific CD8+ T cell immune responses and anti-viral effect of fmDCs and cryoim-mDCs. (**A**) The detection of antigen-crosspresentation on DCs detected by FACS. (**B**) Fold increase of T cell proliferation *ex vivo*. Data are expressed as mean ± SD (error bars). n = 5; ns, not significant by Mann-Whitney U test. (**C**) The detection of T cell activation by the measurement of CD69 expression. (**D**) OVA_257-264_-specific CD8+ T cell detection by tetramers. (**E**) Statistical data of antigen-specific T cell percentage. Data are expressed as mean ± SD; n = 5; *p < 0.05 by Student’s t-test. (**F**) The liver Fluc activity of AdFLO-infected mice monitored by BLI. The immunized mice from various groups were challenged with AdFLO at a dose of 1 × 10^9^ PFU/mouse via tail vein injection five days after the last vaccination. The liver Fluc activity was detected to quantify hepatocellular infection three days after the virus invasion. (**G**) Statistical data of the Fluc activity in mice undergoing different treatments. Statistic data are expressed as mean ± SD; n = 5; *p < 0.05 by one-way ANOVA.

Peptides of OVA_257-264_ were added in the medium during the process of DC maturation, and then the antigen-pulsed fmDCs or cryoim-mDCs were stained by PE-labeled anti-H-2Kb (SIINFEKL) antibody to recognize the OVA antigen presented on the surface of DCs. Data showed that both fmDCs and cryoim-mDCs could be detected with presented antigens on cell surface, but the antigen-positive cell percentage of cryoim-mDCs was slightly lower than that of fmDCs (Fig. 6A). To compare their capacity to stimulate T cells *ex vivo*, we then co-cultured fmDCs or cryoim-mDCs with OT1-transgenetic mice derived CD8+ T cells at a“DCs: T cells” ratio of 1:5 and 1:20, respectively. After three days’ incubation, we counted total T cell number in the co-culture (Fig. 6B) and also detected the activation marker of CD69 on them (Fig. 6C). Data showed that there were no significant differences between fmDCs and cryoim-mDCs in promoting T cell proliferation or activation at the higher incubation ratio of 1:5. However, either T cell proliferation or activation induced by fmDCs was higher than that induced by cryoim-mDCs when they incubated at the lower incubation ratio of 1:20. We next *i.v.* injected those DCs into mice to examine their *in vivo* T cell initiation and anti-viral therapeutic effect. Mice were immunized twice at the 0 and 14 day, and were euthanized 5 days after the last vaccination. Then lymph cells were isolated from the spleen and LLNs, and the OVAp-specific CD8+ T cell response was measured by tetramers (Fig. 6D and E). The percentage of OVAp-specific CD8+ T cells detected in the spleen and LLNs of fmDC-immunized mice was 1.44 ± 0.15% and 4.02 ± 0.21%, respectively. By contrast, the numbers were significantly lower in mice immunized with cryoim-mDCs, with a positive percentage of 0.97 ± 0.07% in the spleen and 0.37 ± 0.09% in the LLNs.

The significantly lower OVAp-specific CD8+ T cells induced by cryoim-mDCs promoted us to test whether their protective capacity against viral invasion was affected. A recombinant adenoviral vector of serotype 5 (AdFLO) bearing the genes for Fluc and OVA_257-264_ was used to mimic liver infection. Intravenous injection of the adenoviral vectors leads predominantly to liver infection. According to the previous report, the measurement of luciferase expression by *in vivo* BLI allowed the quantification of hepatocellular infection with recombinant adenovirus^16^. Mice were immunized twice at the 0 and 14 day, and were infected with the AdFLGO five days after the last vaccination. BLI detection (Fig. 6F and G) showed the AdFLO viral load was dramatically lower in mice immunized with fmDCs than that in mice immunized with cryoim-mDCs or naïve DCs, suggesting that the immuno-protective capacity of cryoim-mDC was significantly reduced compared to its fresh counterpart.

## Discussion

To date, several studies have reported the effect of cryopreservation on antigen-loaded or unloaded matured DCs with results showing that the freezing and thawing process does not interfere with their phenotype *in vitro* as well as the trafficking and functional properties *in vivo*^11^,^17^. However, in contrast to the validate and consistent findings in cryopreserved mature DCs, systematical investigations on the cellular changes and the *in vivo* functional properties of frozen immature DC as well as its mature counterpart are very limited, and some *in vitro* findings are controversial. Lewalle *et al*.^18^ have shown that cryopreservation does not influence imDCs’ ability to induce antigen-specific immune responses or functionally react to maturation stimuli (CD40 ligand and IFN-*γ*). In contrast, John *et al*.^13^ have reported that cryopreservation of imDC results in enhanced cell maturation, reduced endocytic activity and decreased efficiency of adenoviral transduction although the DCs they used were both derived from human.

In this study, the freshly generated imDCs were cryopreserved, revived and matured following the most widely used procedure, with the objective to explore the viability, phenotype, *in vivo* trafficking and therapeutic effects of frozen imDC-derived DC vaccines. We found that more than 90% imDCs could be recovered after the freezing-thawing process without changes of phenotype markers, which were in consistent with the previous findings^15^,^16^ and confirmed that cryopreservation could preserve the viability and biology of imDCs.

In DC-based cell therapy, the delivery routes of DCs play an important role in stimulation of specific T cells and potential immune response. Most commonly DC-based vaccines are injected subcutaneously, intravenously or intradermally. The subcutaneous or intradermal injection is expected to deliver DCs to local LNs. In our study, the most widely used footpad model was employed to examine DC homing capacity to local LNs, with results showing that more than 40% fmDCs could home to LNs, which was about 3-fold more than that of cryoim-mDCs. Different from the way of subcutaneous injection, intravenously injected DCs routinely distribute in a wide range of tissues. The BLI analysis showed that both the fmDCs and cryoim-mDCs accumulated in lungs at 4 h post cell injection, which was in consistent with previous results that the intravenously transferred DCs tended to distributing in lungs at an early time post cell injection, mainly due to mechanical trapping in pulmonary capillaries rather than active adhesion^19^. Our dynamic detection revealed that fmDCs quickly left the lung and finally accessed to and settled in the spleen and LLNs. An interesting finding herein was that the accumulation of fmDCs in LLNs was far more than that in spleens, though the latter are commonly recognized as the lymphoid organ for *i.v.* injected DC homing^20^. We speculate the particular physiological position that LLNs located might be the major reason for circulating DCs preferential homing to this lymph node; that is, they accumulated in livers at the early time of injection (24 and 48 h) and then migrated to LLNs following the lymphatic vessels. In contrast to fmDCs’ transit stay in the lung and liver, a large fraction of injected cryoim-mDCs were still detained in the lung and liver at 72 h, which has never been reported before.

Our research showed that the migration of cryoim-mDCs to lymphoid tissues, by either subcutaneous or intravenous way of delivery, was significantly lower than that of fmDCs. The term “migration” as discussed here, encompasses several strictly regulated discrete events that invoke numerous context-specific cellular and molecular mechanisms. DCs express specific adhesion molecules and maturation-dependent chemoattractant receptors that allow them to respond to a variety of ligands, which control their trafficking. DCs utilize specific chemokine receptor-ligand pathways, such as CCR1-CCL13, CCR2-CCL2, and CXCR1-CXCL8 to navigate within non-lymphoid peripheral tissues^21^,^22^. When DCs receive the “leaving signals”, they down-regulate their responsiveness to these inflammatory chemokine pathways and traffic to the draining LNs by up-regulating CCR7, which responds to two ligands, CCL19 and CCL21 that are expressed by peripheral lymphatic endothelial cells and guide DCs to downstream LNs^23^. Compared to fmDCs, cryoim-mDCs demonstrated significantly higher expressions of CCR1, CCR2, and CXCR1 whereas the expression of CCR7 was significantly lower on them. So the reduced homing ability of cryoim-mDCs was highly likely mediated by the reduced expression of CCR7, and the relatively higher expression of the CCR1 and CCR2 and CXCR1 may also attribute to this process by enhancing the anchors to extracellular matrix proteins. To figure out why CCR7 expression on cryoim-mDCs was lower than that on fmDCs, we detected the secretion of Th1 inflammatory cytokines when fimDCs and cryoimDCs stimulated by LPS (Figure S6 in Supplementary Materials). Data showed that the content of both TNF-*α* and IL-1*β* in the cultural supernatant of fimDCs was significantly higher than that in the culture of cryoimDCs, suggesting the maturation stimuli fmDCs got was more intense than cryoim-mDCs did. Another indispensable factor for the motility of DCs is the dynamics of the cytoskeleton, which can be viewed as “motors” for the chemotaxis of DCs. DCs that failed to regulate F-actin localization and polarity show defects in migration toward chemotactic factors^24^. In this study, we found the freezing and thawing process disturbed the cytoskeleton rearrangement of cryoimDCs upon LPS treatment.

Although the antigen presentation ability of cryoim-mDCs was lower than fmDCs, we didn’t find obvious differences between them in either promoting T cell proliferation or activation *ex vivo* at the higher ratio of “DCs: T cells” (1:5). However, when they were co-incubated in a lower ratio (1:20), T cell activation ability of DCs was positively related to their antigen presentation efficiency, suggesting the high radio of “DCs: T cells” co-culture may cover up the influence exerted by the antigen presentation efficiency of DCs on T cell induction. The observation that cryopreservation didn’t affect T cell activation of DCs at high incubation ratio (1:5) *in vitro* was, however, different from the findings of Silveira *et al*.^25^ and lutz *et al*.^26^. The difference may be caused by the different experimental system that performed. They used the allogeneic lymphocytes (MLR) to evaluate the stimulatory effects of DCs. By contrast, the purified OT1-transgenetic mice derived syngeneic CD8+ T cells were used in our study to better mimic the DC-T cell interaction in physiological state. In addition, the discrepancy of cell-specie we used (murine *vs*. human) may also be one of the possible reasons for the differences.

The *in vivo* antigen-specific CD8+ T cell responses induced by DCs were detected by OVA-specific tetramers. That we choose to detect T cells in LLNs and the spleen is based on the finding that the *i.v.* injected DCs mainly accumulated in these two lymphoid organs. Distinct differences were observed between fmDCs and cryoim-mDCs in initiating the *in vivo* antigen-specific T cell responses in both the spleens and LLNs. We speculate the significantly lower T cell-activation ability of cryoim-mDCs was highly associated with the limited homing cells in these two lymphoid organs. In addition, the relatively lower antigen-presentation of cryoim-mDCs might also be one of the major factors resulting in the weak T cell induction especially at the lower “DCs: T cells” ratio caused by the hindered homing ability of cryoim-mDCs. Our data showed that the anti-viral effect of cryoim-mDCs was significantly lower than that of fmDCs, providing confirming evidence to the widely accepted concept that the homing ability is critically important for DC vaccines to mediate the therapeutic effects.

In conclusion, we focused our study on investigating the effects of freezing-thawing process on DC vaccines’ *in vivo* migration and distribution pattern with a novel finding that the homing ability of cryoim-mDCs, administrated by either subcutaneous or intravenous way, was significantly lower than that of fmDCs. In addition, we also found that the homing ability of DCs was directly correlated with the intensity of *in vivo* T cell responses induced, which was the main reason for the reduced therapeutic effect of cryoim-mDC vaccines.

## Materials and Methods

### Mice

Male wild-type C57BL/6 mice, 5–6 weeks of age were obtained from the Academy of Military Medicine Science animal center. L2G85 (FVB) mice expressing Firefly luciferase (Fluc) were backcrossed into C57BL/6 mice and used at stage N7 (L2G85.C57BL/6).

All experiments were subjected to review and approval of the Animal Care and Use Committee of Academy of Military Medical Sciences (approve number: IACUC of AMMS-09-2015-003). Mice were housed and handled in accordance with the guidelines of the National Institutes of Health.

### Reagents and antibodies

RPMI 1640 was purchased from Life Technologies Corporation (Grand Island, NY, USA), containing 4.5 g/L D-Glucose, L-Glutamine and 110 mg/L Sodium Pyruvate. GM-CSF, IL-4 and TNF-*α* were purchased from Peprotech Asia (Revohot, Israel). Lipopolysaccharide (LPS) was purchased from Sigma Aldrich (Steinheim, Sweden). D-luciferin was purchased from Promega Corporation (Madison, WI, USA). The following antibodies were used for FACS analysis: fluorescein isothiocyanate (FITC)-conjugated anti-CD86, FITC-conjugated anti-I-A[b], FITC-conjugated anti-H-2Kb, Phycoerythrin (PE)-conjugated anti-CCR7, PE-conjugated anti-mouse CD40, PE-conjugated anti-CD11c and FITC conjugated anti-CD11c were purchased from BD PharMingen (SanDiego, CA). PE-conjugated anti-H-2Kb (SIINFEKL) was purchased from BioLegend (San Diego, CA). PE-conjugated anti-CCR1, PE-conjugated anti-CCR2, PE-conjugated anti-CXCR1 and PE-conjugated anti-CCR7 were purchased from R&D Systems (Minneapolis, MN, USA). All the isotype control antibodies were purchased from the same company as the detecting antibodies.

### Generation of mouse bone marrow (BM)-derived DCs

After euthanasia, 5–6 week old L2G85.C57BL/6 mice were sacrificed to collect the femurs by cutting tibia below the knee joints and the pelvic bone close to the hip joint. Muscles adhere to the bone were removed carefully, and the femurs were placed into a dish containing cold RPMI 1640 with 10% FBS. Bone marrow mononuclear cell were extruded by flushing with a syringe filled with medium, following gentle dispersion by pipet. Then, the cell suspensions were cultured at a density of 2 × 10^6^ cells/mL in 6-well plates in RPMI1640 medium supplemented with 10% FBS, 10 ng/mL recombinant murine GM-CSF and 5 ng/mL recombinant murine IL-4, before that the erythrocyte in the cell suspensions were depleted using RBC Lysis Buffer (Cwbio). Old medium was replaced by 2 mL fresh medium with GM-CSF and IL-4 at day 3 and 5. The loosely adherent clusters were used on day 7 as imDCs. mDCs can be generated from imDCs by incubated with LPS (1 *μ*g/mL) for 48 h.

### Cryopreservation and thawing of DCs

DCs were cryopreserved with a common method for cell cryopreservation using a Cryo 1 °C freezing container filled with isopropyl alcohol, which can achieve a 1 °C/min cooling rate at −70 °C for 12–14 h (Nalgene, Nalge Nunc International, Rochester, NY). DCs for cryopreservation were resuspended at a cell concentration of 5 × 10^7^ cells/mL in 90% FBS supplement with 10% dimethyil-sulfoxide (DMSO), then they were aliquoted into cryovials (1 mL per vial) and put into the freezing container at −70 °C for 24 h, finally, they were transferred into liquid nitrogen tank for one month. Cryopreserved DCs were rapidly thawed in 37 °C water bath and then diluted by adding 10 volumes of growth medium and centrifuged at 1200 rpm for 5 min before finally be resuspended in growth media.

### DC phenotyping and apoptosis assay

For phenotyping, the four types of DCs (fimDCs, fmDCs, cryoimDCs and cryoim-mDCs) were washed twice with cold PBS and then re-suspended in Cell Stain Buffer (BioLegend) at a density of 1 × 10^7^ cells/mL. APC-anti-CD11c combined with one of the phenotypeing antibodies (FITC-anti-I-A[b], FITC-anti-H-2Kb, PE-anti-CD40, FITC-anti-CD86) were added to cell suspension at a concentration of 1.0 *μ*g per million cells in 100 *μ*L volume, and then stained half an hour at 4 °C protecting from light. After staining, cells were washed two times with cold PBS and then re-suspended in 4% paraformaldehyde. The double-labeled samples were subjected to FACS analysis in an 8-color FACS Calibur (BD Biosciences, Mountain View, CA). The apoptosis detection kit (FITC Annexin V Apoptosis Detection Kit I, BD PharMingen) was used for the viability analysis. fimDCs and cryoimDCs were washed twice with cold PBS and then re-suspended in 1 × Binding Buffer at a concentration of 1 × 10^6 ^cells/mL. Add 5 *μ*L of FITC Annexin V (0.2 mg/ml) and 5 *μ*L PI (50 *μ*g/mL) per 100 *μ*L cell suspension. Gently vortex the cells and incubate for 15 min at RT (25 °C) in the dark. After that, 400 *μ*L 1× Binding Buffer was added to the incubation system and then the labeled cells were analyzed by FACS Calibur within 1 hour. Total cell populations in FACS analysis were determined by light scatter properties with the purpose to include most of cells and excluded the debris; the population of DCs was defined by CD11c positive population; and the positive population was gated by appropriate isotype controls. Data were collected for 10000 cells and analyzed using Flow Jo 7.6.1 software.

### Immunofluorescence staining and confocal microscopy

1 × 10^6 ^fmDCs or cryoim-mDCs cells ready for detection were adhered on a poly-L-lysine-coated microscope slide at 37 °C for 0.5 h, fixed with 4% paraformaldehyde (Merck, Schwalbach, Germany) for 1 h, and permeabilized with 0.1% Triton X-100 (PBST, Sigma) in PBS for 2 min at room temperature. For F-actin staining, permeabilized cells were labeled with 300 *μ*L FITC-conjugated phalloidin (50 *μ*g/mL) for 1 h at room temperature, followed by washing. For *β*-tublin staining, permeabilized DCs were stained with 300 *μ*L anti-*β*-tublin (10 *μ*g/mL) at 4 °C overnight, followed by washing and the addition of 300 *μ*L Cy3-Goat Anti-Mouse IgG (2 *μ*g/mL) for 2 h at room temperature. For vimentin staining, permeabilized DCs were stained with 300 *μ*L anti-vimentin (1 *μ*g/mL) at 4 °C overnight, followed by washing and the addition of 300 *μ*L 488-Alex-Goat Anti-Rabbit IgG (10 *μ*g/mL) for 2 h at room temperature. After washing, all coverslips were mounted using mounting medium with DAPI and viewed on a confocal laser-scanning microscopy (Zeiss, LSM510META). Triton X-100, phalloidin and DAPI were purchased from Sigma Aldrich (Steinheim, Sweden). The antibodies of anti-*β*-tublin and anti-vimentin and the corresponding secondary antibody were purchased from Abcam (UK Cambridge).

### Chemotaxis

Chemotaxis of fmDCs or cryoim-mDCs was assessed by their migration through an 8 *μ*m polycarbonate membrane (Corning, NY, USA) using CCL19 as the chemoattractant. DCs were seeded (5 × 10^4^ cells in 100 *μ*L of RPMI 1640) in the upper chamber in 24-well cell culture plates and the lower chamber contained 500 *μ*L of RPMI 1640 with or without CCL19 (final concentration, 500 ng/mL). Each condition was set up in three repeats. The plates were incubated for 4 h at 37 °C in 5% CO_2_. The cells remaining in the upper chamber were removed and the migrated cells were harvested by washing the bottom side of the inserts and the wells 3 times. The total migrated cells were counted by use of Cell Counter (IC1000, Countstar, Shanghai, China) and the results were expressed as the mean number of migrating cells ± standard deviation (SD).

### Bioluminescence imaging

We performed imaging 5 min after intraperitoneal injection of D-luciferin (150 mg kg^−1^). The acquisition of image data and measurement of signal intensity were obtained through region of interest analysis on an IVIS Spectrum system (PerkinElmer, Inc.) with Living Image software (Xenogen). For the examination of DC homing to local LNs, a total of 1 × 10^6^ Fluc^+^ DCs were injected subcutaneously into the hind leg footpad that was pretreated with 30 ng TNF-*α* 24 h before cell injection. For DC distribution analysis, 3 × 10^6^ Fluc^+^ DCs were intravenously injected into C57BL/6J mice and imaged at 4, 24, 48 and 72 h after cell injection. For DC tissue bio-distribution analysis, mice were injected intraperitoneally with D-luciferin and euthanized 10 min later. Tissues were dissected and arranged on a cell culture plate or black paper, covered with D-luciferin (3 mg/mL), and imaged for 1 min to obtain photographs.

### Antigen-presenting analysis

Cells were harvested by centrifugation at 200 g for 15 min after being pulsed with 8-mer peptides derived from chicken ovalbumin (OVA)-sequence SIINFEKL(4 *μ*g/mL) for 48 h, then, they were stained with anti-H-2Kb (SIINFEKL)-PE for 15 min at 4 °C. The reaction was stopped by washing twice with PBS and the cells were analyzed by flow cytometer.

### Antigen-specific T cell activation by DC

For the detection of *ex vivo* T cell activation, fmDCs or cryoim-mDCs were co-cultured with OT1-transgenetic mice derived CD8+ T cells at a target and effector ratio of 1:5 and 1:20. After three days’ incubation, we counted total T cell number in the co-culture by automated cell counter and FACS were employed for the detection of the activation marker of CD69 on them. For the detection of the *in vivo* T cell activation, mice were immunized twice, on days 0 and 14, as described in the Figure captions. A total of 3 × 10^6^ Bone Marrow Dendritic Cells (BMDCs) were intravenously administered in DC-treated mice. The mice were euthanized three days after the last vaccination, and LLNs and spleen were separated. Cell suspensions were then stained by antibodies against CD8*α* and H-2kb tetramer-SIINFEKL (MBL, Nagoya, Japan) and then subjected to FACS analysis to distinguish OVAp-specific CD8+ T cells.

### Recombinant Adenovirus Generation and Mouse Infection

Recombinant adenovirus (AdFLO) expressing fusion proteins of the H2-Kb-binding peptide epitope OVA_257-264_ SIINFEKL and Fluc were generated by the AdEasy system. Mice were challenged with AdFLGO at a dose of 1 × 10^9^ PFU/mouse via tail vein injection five days after the last vaccination. The Fluc activity of the mice was monitored by BLI three days after the virus invasion.

### Statistical analysis

Data are presented as mean ± SD. Student’s t-test, one-way one-way ANOVA and Mann-Whitney U test were used for statistical analysis. Significance was accepted at a *P* value of less than 0.05. Computer software (Sigma Plot software, Version 12.0, Systat Software Inc., San Jose, California) was used to perform the statistical analysis.

## Additional Information

**How to cite this article**: Zhou, Q. *et al*. Mature dendritic cell derived from cryopreserved immature dendritic cell shows impaired homing ability and reduced anti-viral therapeutic effects. *Sci. Rep.* **6**, 39071; doi: 10.1038/srep39071 (2016).

**Publisher's note:** Springer Nature remains neutral with regard to jurisdictional claims in published maps and institutional affiliations.

## Supplementary Material

Supplementary Information
