# Supplementary material for: Mature dendritic cell derived from cryopreserved immature dendritic cell shows impaired homing ability and reduced anti-viral therapeutic effects
Source: Sci Rep. 2016 Dec 13;6:39071. doi: 10.1038/srep39071 (PMC5153632; doi:10.1038/srep39071)
Supplement: Supplementary Information [file srep39071-s1.pdf]

Supplementary Materials

Mature dendritic cell derived from cryopreserved immature dendritic cell shows impaired homing ability and reduced anti-viral therapeutic effects

Qianqian Zhou,[a,+] Yulong Zhang,[a,+] Man Zhao,[a] Xiaohui Wang,[a,b] Cong Ma,[c] Xinquan Jiang,[c] Tao Wu,[d] Donggen Wang,[a,]* Linsheng Zhan[a,]*

[a]: Beijing Institute of Transfusion Medicine, Beijing key Laboratory of Blood Safety and Supply Technologies, Beijing 100850, P.R. China.

[b]: Key Laboratory of Advanced Energy Materials Chemistry (Ministry of Education), Nankai University, Tianjin 300071, China.

[c]: School of Public Health, Taishan Medical University , Taian, Shandong 271000, China.

[d]: Department of Blood Transfusion, PLA Army General Hospital, Beijing 100700, P.R. China

*: Corresponding author at: Beijing Institute of Transfusion Medicine, 27(9) Tai Ping Road, Beijing, China, 100850.

E-mail address: lszhan91@yahoo.com; David_king@139.com.

[+] These authors contributed equally to this work.

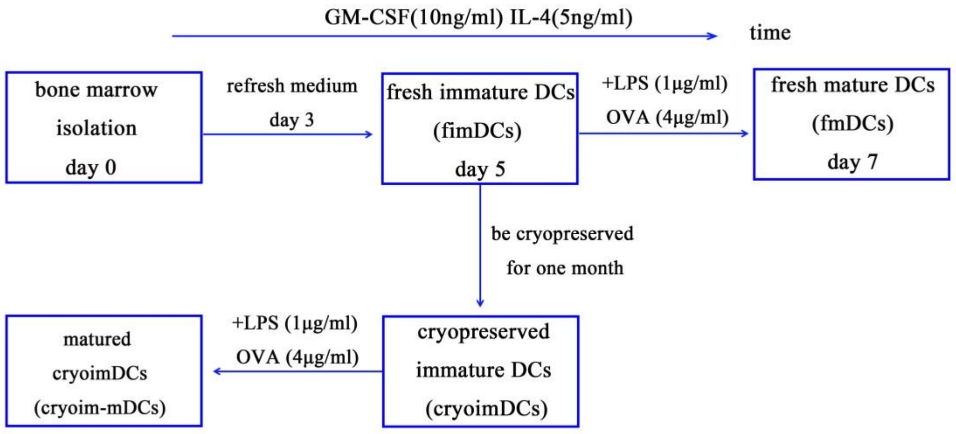

Figure S1. Schematic overview of the differentiation and stimulation protocol. Bone marrow from C57BL/6 was cultured with GM-CSF and IL-4 for 5-7 days to get fimDCs. Parts of cells were

cryopreserved for as long as one month and termed as cryoimDCs. Both fimDCs and cryoimDCs were matured by LPS to get fmDCs and cryoim-mDCs.

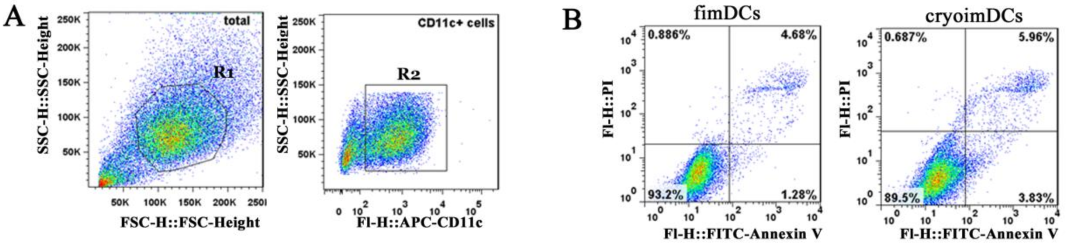

Figure S2. Cryopreservation has little effect on imDCs' viability. (A) Total cell populations (R1) and DCs population (R2) in FACS analysis were determined by the light scatter properties and CD11c fluorescence intensity, respectively. (B) imDCs before and after cryopreservation were doubly stained with PI and Annex-v-FITC and then detected by flow cytometry, of which the cell population with fluorescence double negative represented DCs of high viability.

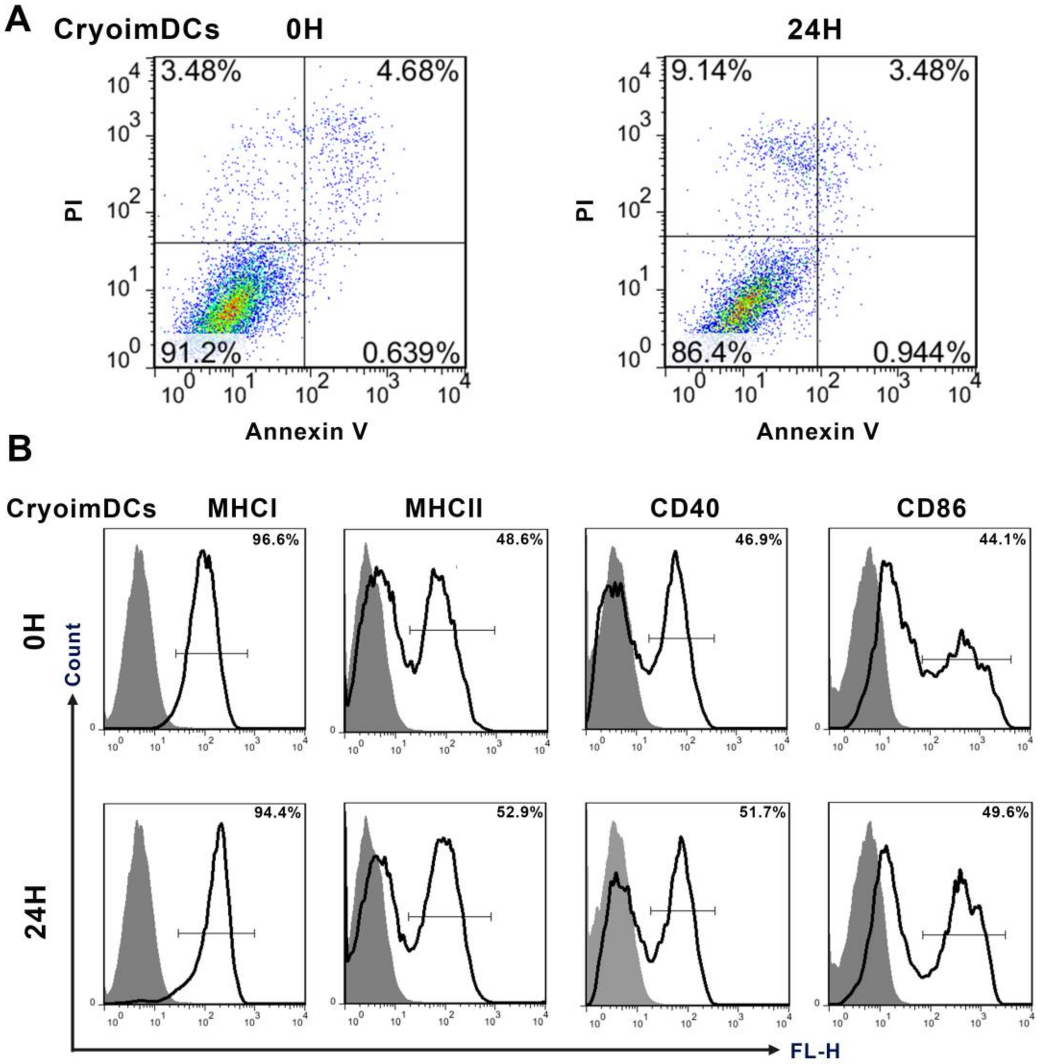

Figure S3. Comparing the viability (A) and surfance marker expression (B) of cyroimDCs between 0 h and 24 h after the thaw of the cells.

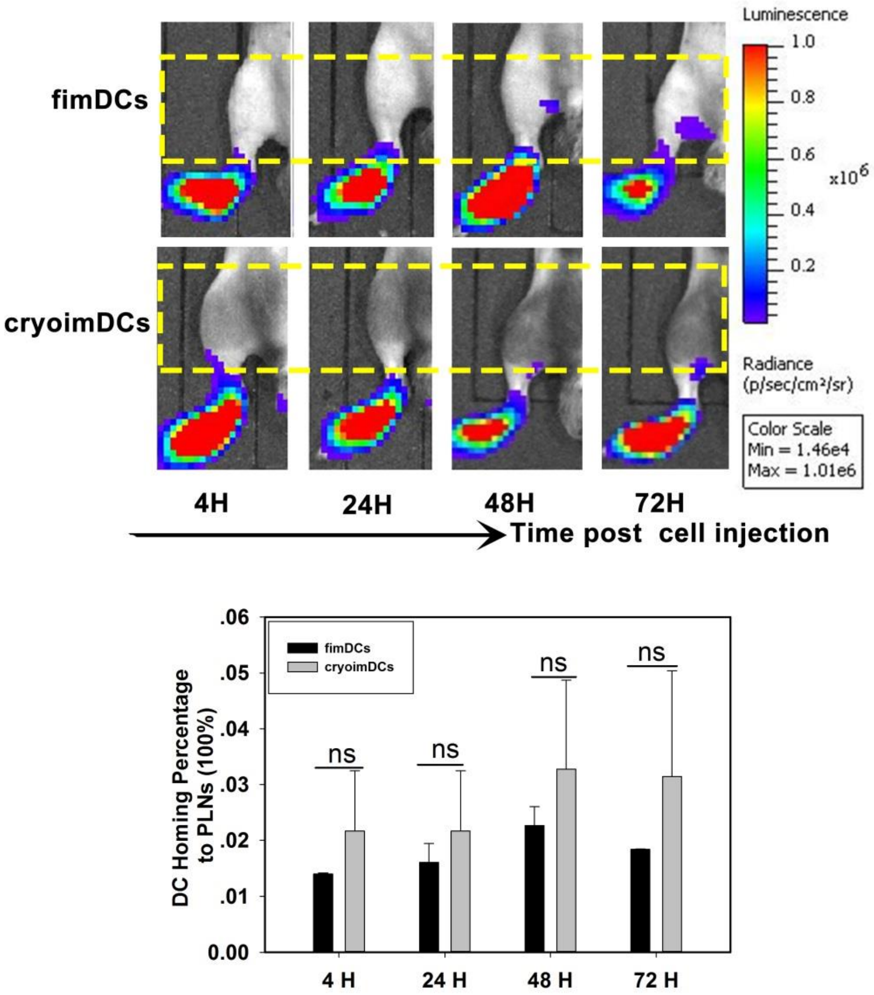

Figure S4. Comparing the homing ability of subcutaneously injected fimDCs and cryoimDCs. The dynamic homing process of fimDCs and cryoimDCs were imaged successively at 4, 24, 48 and 72 h to reflect cells' dynamic migration process, and cell percentage homing to PLNs were quantified by Living Imaging software. Results are representative of two independent experiments. Data are expressed as mean ± SD (error bars). n = 5; ns, not significant.

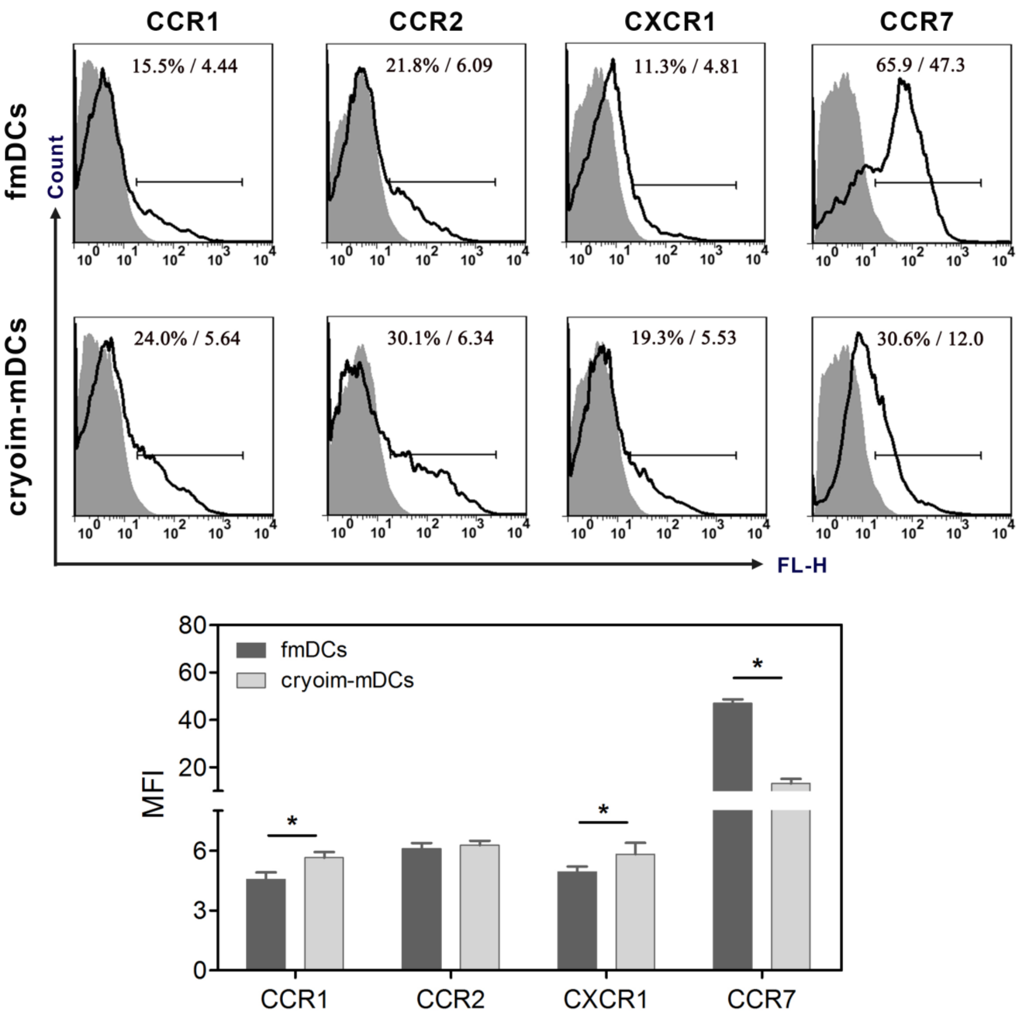

Figure S5. The detection of the MFI changes of CCR1, CCR2, CXCR1 and CCR7 expression on fmDCs and cryoim-mDCs by FACS.

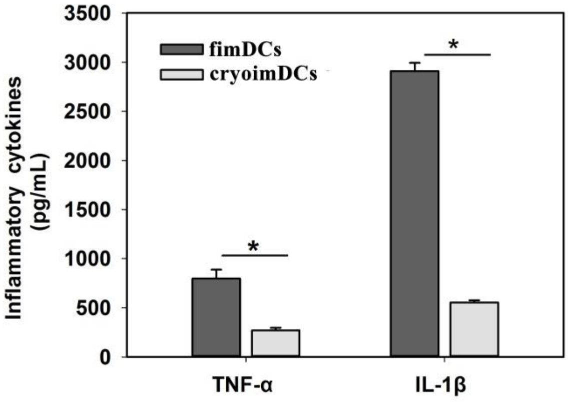

Figure S6. Cryopreservation hindered the cytokines secretions of imDCs. fimDCs and cryoimDCs were cocultured with 1 μg/mL LPS for 48 h and the supernatants were collected to assess for the

presence of IL1-*β* and TNF-*α* by ELISA. Data are expressed as mean ± SD (error bars); n = 5; *$p < 0.05$.

Table S1. List of the tissues that investigated in this work.

Symbol	Tissue	Abbreviation	Location
**a**	Inguinal lymph nodes	ILN	In the groin area, near the deep circumflex iliac artery.
**b**	Axillary lymph nodes	ALN	At the junction between the lateral thoracic vein and the axillary vein.
**c**	Cervical lymph nodes	CLN	Close to the salivary glands (mandibular and superficial parotid lymph nodes).
**d**	Liver lymph nodes	LLN	Located to the right of the portal vein and one was more superficial, while the other was located much deeper in the peritoneal cavity.
**e**	Liver		Whole tissue in the upper quadrant of the abdominal cavity.
**f**	Spleen		Whole tissue in the left quadrant of the abdomen.
**g**	Mesenteric lymph nodes	MLN	In the mesentery, connected to the small intestine.
**h**	Intestine		2-3 inches, emptied of its content.
**i**	Kidney		Pooled two Kidneys in the abdominal cavity.
**j**	Heart		Whole tissue in the chest.
**k**	Lung		Whole tissue in cavities on side of the heart.
